# A comprehensive review of *Mycoplasma pneumoniae* infection in chronic lung diseases: recent advances in understanding asthma, COPD, and bronchiectasis

**DOI:** 10.3389/fmed.2024.1437731

**Published:** 2024-09-25

**Authors:** Zai-qiang Guo, Shun-yi Gu, Zhi-hua Tian, Bo-ying Du

**Affiliations:** ^1^Department of Science and Education, Beijing Fengtai Hospital of Integrated Traditional Chinese and Modern Medicine, Beijing, China; ^2^Department of Internal Medicine, Beijing Tongzhou District Integrated Traditional Chinese and Modern Medicine, Beijing, China; ^3^Department of Science and Education, Beijing Daxing District Hospital of Integrated Traditional Chinese and Modern Medicine, Beijing, China; ^4^Pediatrics, Shijiazhuang Second Hospital, Shijiazhuang, China

**Keywords:** asthma, lung diseases, COPD, pneumonia, *Mycoplasma*

## Abstract

This review summarizes the research progress over the past 30 years on the relationship between *Mycoplasma pneumoniae* infection and chronic respiratory diseases such as asthma, chronic obstructive pulmonary disease (COPD), and bronchiectasis. *Mycoplasma pneumoniae* is a common cause of community-acquired pneumonia, particularly in children and young adults. Key findings from recent studies indicate that *M. pneumoniae* infection is associated with a higher risk of asthma exacerbations and may contribute to the development of bronchiectasis in susceptible individuals. Additionally, emerging evidence suggests that *M. pneumoniae*-induced immune dysregulation plays a crucial role in the pathogenesis of chronic lung diseases. This review aims to summarize the current understanding of the potential links between *M. pneumoniae* pneumonia and various chronic respiratory conditions, including asthma, chronic obstructive pulmonary disease (COPD), and bronchiectasis. We discuss the epidemiological data, pathogenic mechanisms, clinical manifestations, and long-term consequences of *M. pneumoniae*-related respiratory illnesses. Additionally, we highlight the challenges in diagnosis and treatment, as well as future research directions in this field.

## Introduction

*Mycoplasma pneumoniae* is increasingly recognized as a significant factor in the development and exacerbation of chronic lung diseases. While traditionally known as a common cause of community-acquired pneumonia, recent evidence suggests that *M. pneumoniae* infection may have far-reaching consequences on respiratory health (1, 2). This review focuses on the association between *M. pneumoniae* pneumonia and the development or worsening of asthma, chronic obstructive pulmonary disease (COPD), and bronchiectasis.

*Mycoplasma pneumoniae* is a small, cell wall-deficient bacterium belonging to the class Mollicutes, characterized by its ability to adhere to and invade respiratory epithelial cells (3). Despite its minute size and lack of a rigid cell wall, *M. pneumoniae* possesses a remarkable array of virulence factors that enable it to colonize and persist within the respiratory tract, evade host immune defenses, and induce inflammatory responses (4).

The pathogenesis of *M. pneumoniae* respiratory infections is multifaceted, involving both direct cytotoxic effects on host cells and indirect mechanisms that dysregulate the host’s immune responses (5). The adherence and invasion of *M. pneumoniae* to respiratory epithelial cells can lead to cell injury, disruption of the epithelial barrier, and impairment of mucociliary clearance (6). Additionally, *M. pneumoniae* can induce the release of various pro-inflammatory cytokines and chemokines, triggering an exaggerated inflammatory response and contributing to airway hyperresponsiveness and remodeling (7).

While *M. pneumoniae* infections are typically self-limiting in healthy individuals, they can have more severe consequences in certain populations, such as those with underlying respiratory conditions or compromised immune systems (8). The interplay between *M. pneumoniae* and chronic lung diseases is an area of increasing research interest, as emerging evidence suggests that this atypical bacterial pathogen may play a significant role in the development, exacerbation, and progression of various respiratory disorders. We focus on *M. pneumoniae* due to its unique characteristics that enable evasion of host defenses and antibiotics, potential long-term effects on respiratory health beyond acute infections, and the increasing prevalence of antibiotic-resistant strains. This review is the first to comprehensively synthesize recent findings on the multifaceted relationship between *M. pneumoniae* infection and various chronic lung diseases, integrating epidemiological data, pathogenic mechanisms, clinical manifestations, and emerging therapeutic approaches.

## Asthma

Several epidemiological studies have reported a higher prevalence of *M. pneumoniae* infection among individuals with asthma compared to non-asthmatic controls (9, 10). Furthermore, *M. pneumoniae* has been implicated as a contributing factor in asthma exacerbations, with increased hospitalizations and emergency department visits observed during outbreaks. The underlying mechanisms by which *M. pneumoniae* may contribute to asthma pathogenesis and exacerbations are not fully understood but are thought to involve a combination of direct epithelial injury, mucus hypersecretion, and the induction of airway inflammation and hyperresponsiveness (11).

## Chronic obstructive pulmonary disease (COPD)

*Mycoplasma pneumoniae* has also been associated with acute exacerbations of COPD, with several studies reporting its presence in a significant proportion of COPD exacerbations (12). These exacerbations are often characterized by increased dyspnea, cough, and sputum production, and can lead to accelerated lung function decline and disease progression (13). The mechanisms by which *M. pneumoniae* contributes to COPD exacerbations are not well-defined but may involve airway inflammation, impaired mucociliary clearance, and structural changes in the airways (14).

## Bronchiectasis

Bronchiectasis is a chronic respiratory condition characterized by permanent dilation and structural damage of the bronchi, often resulting from recurrent or persistent respiratory infections (15). Several case reports and studies have documented the development or worsening of bronchiectasis following *M. pneumoniae* pneumonia, particularly in children and adults with underlying conditions such as immunodeficiency or ciliary dyskinesia (16–18). *M. pneumoniae* may contribute to the pathogenesis of bronchiectasis by inducing airway inflammation, mucus hypersecretion, and structural airway damage (19).

Despite the growing body of evidence linking *M. pneumoniae* infections to chronic lung diseases, there are still significant gaps in our understanding of the underlying mechanisms, long-term consequences, and optimal management strategies. This review aims to provide a comprehensive overview of the current research progress in this field, highlighting the epidemiological data, pathogenic mechanisms, clinical manifestations, and potential long-term respiratory outcomes associated with *M. pneumoniae*-related chronic lung diseases. Additionally, we will discuss the challenges in diagnosis and treatment, as well as future research directions that could further elucidate this important area of respiratory medicine.

By elucidating the intricate relationships between *M. pneumoniae* and chronic lung diseases, researchers and clinicians can gain valuable insights into disease pathogenesis, identify potential therapeutic targets, and develop more effective prevention and management strategies. Ultimately, this knowledge can contribute to improved clinical outcomes and quality of life for individuals affected by these debilitating respiratory conditions.

## Epidemiology

### *Mycoplasma pneumoniae* and asthma

The association between *M. pneumoniae* infection and various chronic lung diseases, particularly asthma, COPD, and bronchiectasis, has been increasingly recognized through numerous epidemiological studies conducted across different populations and age groups (20). These studies have not only highlighted the higher prevalence of *M. pneumoniae* infection among individuals with these respiratory conditions but have also provided insights into the potential role of this atypical pathogen in disease exacerbations and long-term respiratory outcomes.

Several large-scale epidemiological studies have consistently reported a higher prevalence of *M. pneumoniae* infection among individuals with asthma compared to non-asthmatic controls (21–23). For instance, a retrospective study by Esposito et al. (24) involving 956 children aged 1–14 years found that the prevalence of *M. pneumoniae* infection was significantly higher in children with asthma (24.6%) compared to those without asthma (8.8%). Furthermore, the study revealed that asthmatic children with *M. pneumoniae* infection had a higher risk of developing severe respiratory complications, such as respiratory failure and pneumonia. Another study by Wood et al. (25) investigated the role of *M. pneumoniae* and *Chlamydia pneumoniae* in children with acute asthma exacerbations. The researchers found that *M. pneumoniae* was detected in 64% of patients with acute asthma, 65% of patients with refractory asthma, and 56% of healthy controls, suggesting a potential contribution to the exacerbation of asthma symptoms.

The association between *M. pneumoniae* epidemics and increased asthma hospitalizations has also been well-documented (26). A study by Sutherland and Martin (27) examined the temporal relationship between *M. pneumoniae* epidemics and asthma hospitalizations in children. The authors reported a strong correlation between *M. pneumoniae* epidemics and increased asthma hospitalizations, highlighting the potential role of this pathogen in triggering asthma exacerbations.

### *Mycoplasma pneumoniae* and COPD

The epidemiological link between *M. pneumoniae* and COPD exacerbations has been explored in several studies (13). Llor and Bjerrum (28) conducted a prospective study involving 311 patients with COPD and found that *M. pneumoniae* was responsible for approximately 10% of acute exacerbations. Furthermore, the study revealed that *M. pneumoniae*-associated exacerbations were more likely to be severe and required hospitalization. In another study by Meloni et al. (29), the authors investigated the role of atypical pathogens, including *M. pneumoniae*, in COPD exacerbations. 17.5% of patients with community-acquired pneumonia and 6.7% of patients with acute COPD episodes were found to be infected with *M. pneumoniae*. Importantly, patients with *M. pneumoniae*-associated exacerbations had a higher risk of severe exacerbations and a longer duration of symptoms compared to those with other respiratory pathogens.

### *Mycoplasma pneumoniae* and bronchiectasis

The epidemiological evidence linking *M. pneumoniae* infection to bronchiectasis has been derived primarily from case reports and smaller studies (30–32). However, these studies have consistently demonstrated a potential role of *M. pneumoniae* in the development or worsening of bronchiectasis, particularly in children and individuals with underlying conditions that predispose them to respiratory infections. A study by Chang and Redding (33) investigated the incidence of post-infection bronchiectasis caused by *M. pneumoniae* in children with asthma. The researchers found that among children with asthma who developed post-infectious bronchiectasis, *M. pneumoniae* was the most commonly identified pathogen, suggesting a significant contribution to the development of this chronic respiratory condition. Similarly, King et al. (34) conducted a retrospective study examining the association between *M. pneumoniae* infection and bronchiectasis in adults. The study identified a subset of patients with non-cystic fibrosis bronchiectasis who had a history of *M. pneumoniae* infection, supporting the potential role of this pathogen in the pathogenesis of bronchiectasis in adults.

While these epidemiological studies have provided valuable insights into the association between *M. pneumoniae* and chronic lung diseases, it is important to note that the interpretation of these findings should be approached with caution. Many of these studies are observational in nature, making it difficult to establish a direct causal relationship. Additionally, the presence of confounding factors, such as co-infections with other respiratory pathogens or underlying comorbidities, may influence the observed associations. To further strengthen the epidemiological evidence, larger-scale, prospective studies with rigorous control for potential confounding factors are needed. These studies should also incorporate detailed clinical and laboratory data, as well as long-term follow-up, to better understand the temporal relationship between *M. pneumoniae* infection and the development or exacerbation of chronic lung diseases.

Overall, the growing body of epidemiological evidence highlights the significant association between *M. pneumoniae* infection and chronic lung diseases, particularly asthma, COPD, and bronchiectasis. It is important to acknowledge their limitations. Sample sizes vary considerably across studies, potentially affecting the reliability and generalizability of results. Geographical differences in *M. pneumoniae* prevalence and strain distribution may also influence findings. Additionally, variations in diagnostic methods and study designs can make direct comparisons challenging. Future epidemiological research should focus on large-scale, multicenter studies with standardized diagnostic approaches to better elucidate the true prevalence and impact of *M. pneumoniae* in chronic lung diseases across diverse populations and regions.

## Pathogenic mechanisms

The pathogenic mechanisms underlying the association between *Mycoplasma pneumoniae* and chronic lung diseases are multifaceted and involve a complex interplay between the bacterial virulence factors, host immune responses, and respiratory tissue damage (35). While the exact mechanisms are not fully elucidated, current research has shed light on several direct and indirect pathways through which *M. pneumoniae* contributes to the development and exacerbation of chronic respiratory conditions.

*Mycoplasma pneumoniae* infection can significantly impact immune function in chronic lung diseases. In asthma, *M. pneumoniae* has been shown to induce a Th2-biased immune response, leading to increased production of IL-4 and IL-5, which contribute to airway hyperresponsiveness and eosinophilic inflammation (36). In COPD, *M. pneumoniae* infection can exacerbate the already dysregulated immune response, leading to increased neutrophilic inflammation and oxidative stress. In bronchiectasis, *M. pneumoniae* may impair mucociliary clearance and innate immune defenses, promoting chronic bacterial colonization and recurrent infections (37).

## Direct effects

*Mycoplasma pneumoniae* possesses specialized adhesive proteins and cytadherence accessory proteins that facilitate its adherence and invasion into respiratory epithelial cells (38). This intimate interaction with host cells is a critical step in the pathogenesis of *M. pneumoniae* infections and can lead to several detrimental effects on the respiratory epithelium.

Upon adherence and internalization, *M. pneumoniae* can induce cytotoxic effects on respiratory epithelial cells through various mechanisms, including the production of cytolytic proteins, the induction of apoptosis, and the generation of reactive oxygen species (39). This cytotoxicity results in epithelial cell damage, disruption of the epithelial barrier integrity, and impairment of mucociliary clearance mechanisms (40).

*Mycoplasma pneumoniae* infection has been shown to stimulate excessive mucus production by respiratory epithelial cells, leading to mucus hypersecretion and airway obstruction (40). This effect is mediated by the ability of *M. pneumoniae* to induce the expression of mucin genes and the activation of signaling pathways involved in mucus production (41).

*Mycoplasma pneumoniae* can trigger potent inflammatory responses in the respiratory tract by activating pattern recognition receptors (PRRs) on host cells, leading to the production of pro-inflammatory cytokines, chemokines, and other mediators (42). This excessive inflammation can contribute to airway hyperresponsiveness, tissue damage, and remodeling processes associated with chronic lung diseases.

## Indirect effects

*Mycoplasma pneumoniae* infection can dysregulate the host’s immune responses, leading to an imbalance between pro-inflammatory and anti-inflammatory mediators (11). This dysregulation may result in persistent airway inflammation, even after the resolution of the acute infection, thereby perpetuating the pathogenic processes underlying chronic lung diseases.

Emerging evidence suggests that *M. pneumoniae* infection may trigger autoimmune responses through molecular mimicry or other mechanisms (43). These autoimmune reactions can target self-antigens in the respiratory tract, leading to sustained inflammation and tissue damage, contributing to the development or exacerbation of chronic lung diseases.

The chronic inflammation induced by *M. pneumoniae* infection can initiate and perpetuate airway remodeling processes, including smooth muscle hyperplasia, subepithelial fibrosis, and angiogenesis (44). These structural changes in the airways can lead to airflow obstruction, impaired lung function, and the progression of chronic respiratory conditions.

In individuals with pre-existing chronic lung diseases, such as asthma, COPD, or bronchiectasis, *M. pneumoniae* infection can act as a trigger for acute exacerbations (45). The inflammatory and tissue-damaging effects of *M. pneumoniae* can exacerbate underlying respiratory conditions, leading to more severe symptoms, lung function decline, and increased disease burden. The pathogenic mechanisms of *M. pneumoniae* are further complicated by the ability of this pathogen to form biofilms, which can enhance its persistence, resistance to antimicrobial agents, and potential for chronic or recurrent infections (46, 47). Additionally, the interplay between *M. pneumoniae* and other respiratory pathogens or environmental factors may contribute to the development and progression of chronic lung diseases through synergistic or additive effects (48).

Understanding the complex pathogenic mechanisms involved in *M. pneumoniae*-associated chronic lung diseases is crucial for the development of effective therapeutic and preventive strategies. Ongoing research efforts are focused on elucidating the intricate molecular and cellular interactions between *M. pneumoniae* and host cells, as well as identifying potential therapeutic targets to mitigate the detrimental effects of this atypical pathogen. Furthermore, the role of host genetic factors, environmental exposures, and comorbidities in modulating the pathogenic mechanisms of *M. pneumoniae* should be explored. By unraveling the intricate interplay between these factors, researchers can gain a more comprehensive understanding of the complex pathways leading to chronic lung diseases and develop tailored interventions for different patient populations.

## Clinical manifestations

The clinical manifestations of *M. pneumoniae*-associated chronic lung diseases can vary substantially depending on the underlying respiratory condition, the severity of the infection, and the age and immune status of the affected individual (49). While some patients may experience acute exacerbations or worsening of pre-existing symptoms, others may develop more insidious or persistent respiratory complaints. Understanding these diverse clinical presentations is crucial for prompt recognition, appropriate management, and prevention of long-term complications.

*Mycoplasma pneumoniae* infection is a well-recognized trigger for acute asthma exacerbations, characterized by increased cough, wheezing, shortness of breath, and airway obstruction (50). These exacerbations can range from mild to life-threatening and often require intensification of bronchodilator and corticosteroid therapy (27). Several studies have reported persistent airway hyperresponsiveness and lung function impairment following *M. pneumoniae* infection in asthmatic individuals (51). This sustained airway hyperresponsiveness may contribute to ongoing respiratory symptoms and increased susceptibility to future asthma exacerbations. *M. pneumoniae* infection has been associated with reduced responsiveness to corticosteroid treatment in asthmatic patients, potentially contributing to more severe and prolonged exacerbations (52). The mechanisms underlying this steroid resistance are not fully understood but may involve the induction of inflammatory pathways that interfere with the anti-inflammatory effects of corticosteroids. Chronic or recurrent *M. pneumoniae* infections in asthmatic individuals have been linked to airway remodeling processes, such as smooth muscle hyperplasia, subepithelial fibrosis, and goblet cell hyperplasia (53). These structural changes in the airways can lead to fixed airflow obstruction and progressive lung function decline.

*Mycoplasma pneumoniae* is a well-documented cause of acute exacerbations in COPD patients, characterized by increased dyspnea, cough, and sputum production (54). These exacerbations can be severe and may require hospitalization, contributing to a significant disease burden and healthcare utilization. Lung Function Decline: Several studies have suggested that *M. pneumoniae*-associated COPD exacerbations may contribute to accelerated lung function decline and disease progression (55). The inflammatory and tissue-damaging effects of *M. pneumoniae* may exacerbate the underlying pathological processes in COPD, leading to more rapid lung function deterioration. *M. pneumoniae* has been identified as a risk factor for increased mortality in COPD patients, particularly during periods of acute exacerbations (56). This increased mortality risk may be related to the severity of the exacerbations, the development of respiratory complications, and the potential for impaired immune responses in COPD patients. In severe cases, *M. pneumoniae*-associated COPD exacerbations can lead to acute respiratory failure, requiring intensive care unit admission and mechanical ventilation (57). This complication is more common in patients with advanced COPD, underlying comorbidities, and severe exacerbations.

*Mycoplasma pneumoniae* infection can trigger acute exacerbations of bronchiectasis, characterized by increased cough, sputum production, and respiratory distress (58). These exacerbations can lead to further lung damage and may require antibiotic treatment and supportive care. *M. pneumoniae* may contribute to the progression of bronchiectasis by causing airway inflammation, mucus hypersecretion, and structural changes in the airways (59). This ongoing airway damage can lead to further bronchial dilation and exacerbate the vicious cycle of infection, inflammation, and airway remodeling. Patients with bronchiectasis and *M. pneumoniae* infection may experience worsening lung function and increased respiratory symptoms, such as dyspnea and exercise intolerance (60). This impairment in lung function can significantly impact quality of life and contribute to the overall disease burden. In severe cases, *M. pneumoniae*-associated bronchiectasis exacerbations can lead to respiratory failure, pneumonia, or other life-threatening complications, particularly in individuals with underlying immunodeficiency or advanced bronchiectasis (61).

In addition to the clinical manifestations in asthma, COPD, and bronchiectasis, *M. pneumoniae* infection has been associated with various other respiratory presentations, including: organizing pneumonia (62), pleurisy and pleural effusions (63), pulmonary nodules and infiltrates (64), and cough and respiratory distress (65).

The clinical manifestations of *M. pneumoniae*-associated chronic lung diseases can vary widely, ranging from acute exacerbations to persistent respiratory symptoms and progressive lung function decline. *M. pneumoniae*-induced exacerbations in chronic lung diseases may feature gradual onset, persistent cough, extrapulmonary symptoms, and poor response to beta-lactam antibiotics. However, these are not definitive, and laboratory confirmation is necessary for diagnosis. Hospitalization or prolonged illness in chronic lung disease patients is not solely due to *M. pneumoniae* infection. The underlying lung disease severity significantly influences outcomes. *M. pneumoniae* may trigger exacerbations, but the clinical picture results from interactions between the pathogen, host immune response, and pre-existing condition. Early recognition and appropriate management of these manifestations are crucial to prevent long-term complications and improve patient outcomes. Additionally, understanding the diverse clinical presentations can aid in the development of targeted diagnostic and therapeutic strategies tailored to specific patient populations and disease phenotypes.

## Diagnosis

The accurate and timely diagnosis of *Mycoplasma pneumoniae* infection in patients with chronic lung diseases poses several challenges due to the limitations of currently available diagnostic methods, the potential for co-infections with other respiratory pathogens, and the diverse clinical presentations (66). Nonetheless, establishing a prompt and reliable diagnosis is crucial for initiating appropriate treatment, preventing complications, and improving patient outcomes.

Traditionally, the diagnosis of *M. pneumoniae* infections has relied on culture-based methods, which involve the isolation of the organism from respiratory specimens, such as sputum, nasopharyngeal swabs, or bronchoalveolar lavage fluid (67). However, these culture techniques are time-consuming, laborious, and often lack sensitivity, making them less suitable for routine clinical use, especially in patients with chronic lung diseases.

With the advent of molecular diagnostic techniques, the detection of *M. pneumoniae* has become more sensitive and specific. Nucleic acid amplification tests (NAATs), such as polymerase chain reaction (PCR) and real-time PCR, have emerged as the preferred methods for the rapid and accurate identification of *M. pneumoniae* in clinical samples (68, 69). PCR-based assays can detect *M. pneumoniae* DNA directly from respiratory specimens, providing valuable information about the presence of the pathogen. Additionally, quantitative real-time PCR can estimate the bacterial load, which may aid in differentiating acute infections from chronic or persistent colonization (70). Despite their improved sensitivity and specificity, molecular diagnostic techniques have some limitations. False-negative results can occur, particularly in the later stages of infection when bacterial loads are low or in patients with impaired immune responses (71). Furthermore, the detection of *M. pneumoniae* DNA does not necessarily indicate an active or clinically significant infection, as the organism can persist in the respiratory tract for extended periods (72).

Serological tests, such as enzyme-linked immunosorbent assays (ELISAs), are commonly used to detect antibodies against *M. pneumoniae* in serum or plasma samples (73). These tests can identify both acute and past infections by measuring immunoglobulin M (IgM) and immunoglobulin G (IgG) antibody levels, respectively. ELISAs are widely available, relatively inexpensive, and can provide valuable information about the immune response to *M. pneumoniae* infection. However, they have several limitations, including potential cross-reactivity with other respiratory pathogens, the delayed appearance of detectable antibody levels, and the persistence of antibodies for extended periods, which can make it challenging to differentiate acute from past infections (74).

Complement fixation tests (CFTs) have been traditionally used for the serological diagnosis of *M. pneumoniae* infections (71). These tests detect the presence of antibodies that bind to *M. pneumoniae* antigens and activate the complement cascade. While CFTs offer good specificity, they are labor-intensive, require specialized laboratory facilities, and may lack sensitivity in certain patient populations or stages of infection (75). Additionally, the interpretation of CFT results can be challenging, as antibody levels can remain elevated for prolonged periods, making it difficult to distinguish acute from past infections.

To improve diagnostic accuracy and overcome the limitations of individual testing methods, a combined diagnostic approach is often recommended for the diagnosis of *M. pneumoniae* infections in patients with chronic lung diseases (76, 77). Molecular tests can identify the presence of the pathogen, while serological tests can help differentiate acute from past infections and monitor the immune response, so the combined use of PCR-based methods for the direct detection of *M. pneumoniae* and serological tests for the assessment of antibody responses can provide complementary information (78). In addition to laboratory testing, the diagnostic process should incorporate a thorough evaluation of the patient’s clinical presentation, including respiratory symptoms, physical examination findings, and radiological imaging results (79).

It is essential to consider the possibility of co-infections with other respiratory pathogens, such as viruses, bacteria, or atypical pathogens, which can complicate the diagnosis and management of *M. pneumoniae*-associated chronic lung diseases (80). Multiplex molecular assays or comprehensive respiratory pathogen panels can aid in the simultaneous detection of multiple pathogens, facilitating a more comprehensive diagnostic approach. In certain clinical scenarios or patient populations, specialized diagnostic tests may be warranted. For example, in patients with suspected autoimmune or inflammatory complications related to *M. pneumoniae* infection, testing for autoantibodies or specific inflammatory markers may provide additional diagnostic insights (81, 82).

Despite the advancements in diagnostic techniques, several challenges remain in the accurate and timely diagnosis of *M. pneumoniae* infections in chronic lung diseases. Accurate and timely diagnosis of *M. pneumoniae* infections in patients with chronic lung diseases is essential for initiating appropriate treatment, preventing complications, and improving patient outcomes. While various diagnostic techniques are available, a combined approach that integrates molecular, serological, clinical, and radiological data is often recommended to overcome the limitations of individual methods. Continued research efforts are needed to develop improved diagnostic algorithms, point-of-care testing, novel biomarkers, and comprehensive pathogen panels, ultimately enhancing our ability to accurately diagnose and manage *M. pneumoniae*-associated chronic lung diseases.

## Treatment challenges

The treatment of *M. pneumoniae*-associated chronic lung diseases presents several challenges that must be addressed to ensure optimal patient outcomes and prevent long-term complications. These challenges encompass antibiotic resistance, potential side effects, and the need for long-term management strategies.

*M. pneumoniae* has developed resistance to several commonly used antibiotics, particularly macrolides and fluoroquinolones, which have traditionally been the mainstay of treatment for this pathogen (83, 84). The emergence of antibiotic-resistant strains has been linked to the widespread and often indiscriminate use of antibiotics, as well as the ability of *M. pneumoniae* to acquire resistance through horizontal gene transfer and mutations (85, 86). Macrolide resistance in *M. pneumoniae* is primarily mediated by mutations in the 23S rRNA gene, which can confer high-level resistance to this class of antibiotics (87). Fluoroquinolone resistance, on the other hand, is often associated with mutations in the parC and parE genes, which encode DNA topoisomerase IV subunits (88). The increasing prevalence of antibiotic-resistant *M. pneumoniae* strains poses significant challenges in the effective management of chronic lung diseases, as it limits the available treatment options and may lead to treatment failures or the need for alternative, potentially less effective or more toxic agents (89).

The antibiotics commonly used to treat *M. pneumoniae* infections, such as macrolides and tetracyclines, can have significant side effects, particularly with prolonged use or in certain patient populations (90). These side effects may include gastrointestinal disturbances, hepatotoxicity, photosensitivity, and adverse effects on the developing fetus (91, 92).

In patients with chronic lung diseases, the potential for drug interactions and cumulative toxicity must be carefully considered, as these individuals often require multiple concurrent medications for the management of their underlying conditions (93). Additionally, the use of certain antibiotics may exacerbate or contribute to the development of antibiotic-associated complications, such as *Clostridium difficile* infections or the disruption of the respiratory microbiome (94, 95).

In cases of chronic lung diseases exacerbated by *M. pneumoniae* infection, long-term management strategies may be required to control symptoms, prevent disease progression, and minimize the risk of recurrent infections or exacerbations (96). These strategies may involve a combination of antibiotics, bronchodilators, corticosteroids, and other supportive therapies, depending on the underlying condition and disease severity (97, 98).

However, the optimal duration and regimens for antibiotic therapy in *M. pneumoniae*-associated chronic lung diseases are not well-established, and there is a lack of consensus regarding the most appropriate long-term management approaches (99). Prolonged or repeated courses of antibiotics may increase the risk of antibiotic resistance, adverse effects, and disruption of the respiratory microbiome, potentially leading to unfavorable outcomes (100). In chronic lung diseases, repeated antibiotic exposure may select for resistant strains, potentially causing persistent infections and recurrent exacerbations. These factors, coupled with *M. pneumoniae*’s long-term impact on respiratory health, make its antibiotic resistance particularly challenging in managing chronic lung conditions.

## Long-term consequences

*Mycoplasma pneumoniae* pneumonia can have long-lasting consequences on respiratory health, even after the resolution of the acute infection. These potential long-term effects warrant close monitoring and appropriate management strategies to mitigate the impact on patient outcomes and quality of life.

Several studies have reported persistent lung function abnormalities, including airflow obstruction, reduced diffusion capacity, and decreased exercise tolerance, following *M. pneumoniae* pneumonia (101, 102). These functional impairments may contribute to the development or exacerbation of chronic lung diseases and can have a significant impact on respiratory health and overall well-being.

*Mycoplasma pneumoniae* infection has been associated with structural changes in the airways, such as airway wall thickening, smooth muscle hyperplasia, and subepithelial fibrosis (103, 104). These remodeling processes can lead to airflow obstruction, impaired lung function, and the progression of chronic respiratory conditions, even after the resolution of the initial infection.

Some individuals may experience persistent respiratory symptoms, including cough, wheezing, and dyspnea, even after the resolution of the acute *M. pneumoniae* infection (105, 106). These chronic symptoms can significantly impact quality of life and may require long-term management strategies, such as bronchodilator therapy or pulmonary rehabilitation.

*Mycoplasma pneumoniae* infection may predispose individuals to subsequent respiratory infections, potentially due to impaired immune responses or structural changes in the airways (107, 108). This increased susceptibility can contribute to the development or exacerbation of chronic lung diseases, as recurrent infections can perpetuate the cycle of inflammation, tissue damage, and airway remodeling.

## Future research directions

Despite the growing body of evidence linking *M. pneumoniae* infection to chronic lung diseases, several areas require further investigation to improve our understanding and develop effective management strategies.

Pathogenic mechanisms: Continued research efforts are needed to elucidate the specific molecular and cellular mechanisms by which *M. pneumoniae* contributes to the development and exacerbation of chronic lung diseases (109). This includes exploring the role of host-pathogen interactions, immune dysregulation, and airway remodeling processes, as well as identifying potential therapeutic targets to mitigate the detrimental effects of this atypical pathogen.

Diagnostic tools: The development of more sensitive, specific, and rapid diagnostic tools is crucial for the accurate and timely detection of *M. pneumoniae* infections, particularly in the context of chronic lung diseases (110). Novel molecular and serological approaches, as well as point-of-care testing methods, should be explored to improve diagnostic capabilities and facilitate prompt intervention.

Antimicrobial resistance: Ongoing surveillance and research efforts are necessary to monitor the emergence and spread of antibiotic-resistant *M. pneumoniae* strains, as well as to investigate the mechanisms of resistance and potential strategies to overcome it (111). Additionally, the development of new antimicrobial agents with novel mechanisms of action should be a priority to address the growing challenge of antibiotic resistance.

Vaccination strategies: The development of effective vaccines against *M. pneumoniae* could provide a promising preventive strategy for reducing the burden of *M. pneumoniae*-associated respiratory diseases, including chronic lung conditions (112). Further research is needed to evaluate the safety, immunogenicity, and efficacy of potential vaccine candidates, as well as to explore novel vaccine platforms and delivery methods.

Long-term follow-up studies: Longitudinal studies monitoring the long-term respiratory outcomes of individuals with *M. pneumoniae* infections are essential to understand the potential for chronic lung disease development and progression (113). These studies could provide valuable insights into risk factors, prognostic markers, and optimal management strategies, ultimately improving patient outcomes and quality of life.

Personalized and precision medicine approaches: Integrating genomic, proteomic, and other omics data with clinical and epidemiological data may facilitate the development of personalized and precision medicine approaches for the management of *M. pneumoniae*-associated chronic lung diseases (114). These approaches could enable tailored treatment strategies based on individual patient characteristics, disease phenotypes, and molecular signatures, leading to improved outcomes and more efficient use of healthcare resources.

Genetic factors play a crucial role in determining individual susceptibility to *M. pneumoniae* infection and its impact on chronic lung diseases. Future research should focus on identifying specific gene variations or deletions that may predispose individuals to *M. pneumoniae*-associated chronic lung diseases. For instance, polymorphisms in toll-like receptor (TLR) genes have been associated with increased susceptibility to *M. pneumoniae* infection and more severe clinical outcomes in asthma patients (115). Additionally, variations in genes involved in the innate immune response, such as mannose-binding lectin (MBL) and surfactant proteins, may influence the risk of developing bronchiectasis following *M. pneumoniae* infection (116). Understanding these genetic factors could lead to personalized prevention and treatment strategies for *M. pneumoniae*-associated chronic lung diseases.

By addressing these future research directions, researchers and clinicians can gain a deeper understanding of the complex interplay between *M. pneumoniae* and chronic lung diseases, leading to the development of more effective diagnostic tools, targeted therapeutic interventions, and preventive strategies. Ultimately, these efforts will contribute to improved patient outcomes, reduced disease burden, and enhanced quality of life for individuals affected by these debilitating respiratory conditions.

## Conclusion

This comprehensive review underscores the significant impact of *Mycoplasma pneumoniae* on chronic lung diseases, particularly asthma, COPD, and bronchiectasis. Evidence from epidemiological studies, pathogenic mechanisms, and clinical manifestations highlights *M. pneumoniae*’s role not only in triggering acute exacerbations but also in potentially contributing to disease development and progression. The pathogen’s ability to induce airway inflammation, disrupt epithelial barriers, and modulate immune responses appears central to its effects on respiratory health. However, challenges remain, including the emergence of antibiotic-resistant strains, limitations in current diagnostic methods, and potential long-term respiratory sequelae. Future research should focus on developing more sensitive diagnostic tools, novel therapeutic approaches, and effective vaccination strategies. The recognition of *M. pneumoniae*’s long-term consequences emphasizes the need for prolonged follow-up and personalized management strategies. As our understanding evolves, it is crucial for clinicians and researchers to remain vigilant in diagnosing, treating, and preventing *M. pneumoniae*-associated respiratory illnesses, ultimately improving outcomes for individuals affected by chronic lung diseases.
