# Corrigendum: A comprehensive review of *Mycoplasma pneumoniae* infection in chronic lung diseases: recent advances in understanding asthma, COPD, and bronchiectasis

**DOI:** 10.3389/fmed.2024.1512825

**Published:** 2024-11-08

**Authors:** Zai-qiang Guo, Shun-yi Gu, Zhi-hua Tian, Bo-ying Du

**Affiliations:** ^1^Department of Science and Education, Beijing Fengtai Hospital of Integrated Traditional Chinese and Modern Medicine, Beijing, China; ^2^Department of Internal Medicine, Beijing Tongzhou District Integrated Traditional Chinese and Modern Medicine, Beijing, China; ^3^Department of Science and Education, Beijing Daxing District Hospital of Integrated Traditional Chinese and Modern Medicine, Beijing, China; ^4^Pediatrics, Shijiazhuang Second Hospital, Shijiazhuang, China

**Keywords:** asthma, lung diseases, COPD, pneumonia, *Mycoplasma*

In the published article, there was an error in the Funding statement. The project number was missing. The correct Funding statement appears below.

